# Prevalence and factors associated with cerebral malaria among children aged 6 to 59 months with severe malaria in Western Uganda: a hospital-based cross-sectional study

**DOI:** 10.1186/s12887-024-05178-z

**Published:** 2024-11-06

**Authors:** Banga Mseza, Patrick Kumbakulu Kumbowi, Martin Nduwimana, Desire Banga, Emmanuel Tibasima Busha, Walufu Ivan Egesa, Richard Justin Odong, Grace Ndeezi

**Affiliations:** 1 Department of Pediatrics and Child Health, Faculty of Clinical Medicine and Dentistry, Kampala International University, Kampala, Uganda; 2 Department of Pediatrics and Child Health, Makerere University College of Health Sciences, Kampala, Uganda; 3 Department of Pediatrics and Child Health, Université Libre Des Pays Des Grands Lacs, Goma, Democratic Republic of Congo; 4 Department of Pediatrics, Nile International Hospital, Jinja, Uganda

**Keywords:** Cerebral malaria, Associated factors, Under-five, Uganda

## Abstract

**Introduction:**

Cerebral malaria, caused by Plasmodium falciparum, represents the most severe neurologic complication of malaria. Its association with high morbidity and mortality rates, especially among young children, underscores its clinical significance. In sub-Saharan Africa, including Uganda, cerebral malaria remains a major health challenge, contributing significantly to the high child mortality rate. Despite advances in malaria control, the burden of cerebral malaria among children under five is substantial, reflecting the need for targeted interventions and improved management strategies. This study aimed to determine the prevalence of cerebral malaria and identify associated factors among children admitted with severe malaria at a tertiary hospital in western Uganda.

**Methods:**

This was a cross-sectional, descriptive, and analytical study involving children aged 6 to 59 months admitted with severe malaria. The study was conducted from January to March 2023 at Fort Portal Regional Referral Hospital. Severe and cerebral malaria were defined as per the WHO criteria. Sociodemographic, clinical, and laboratory data were collected and analyzed using IBM SPSS version 27. Logistic regression analysis was used to evaluate the factors associated with cerebral malaria. A *p*-value < 0.05 indicated statistical significance.

**Results:**

A total of 250 children were recruited (mean age 33.1 ± 17.3 months). The prevalence of cerebral malaria was 12.8% (95% CI: 8.9–17.6). Cerebral malaria was independently associated with male sex (aOR: 3.05, 95% CI: 1.20–7.77, *p* = 0.02), abnormal bleeding (aOR: 13.22, 95% CI: 11.54–15.16, *p* = 0.001), history of convulsions (aOR 12.20, 95% CI: 10.7–21.69, *p* = 0.010), acute kidney injury (aOR: 4.50, 95% CI: 1.30–15.53, *p* = 0.02), and hyponatremia (aOR: 3.47, 95% CI: 1.34–8.96, *p* = 0.010).

**Conclusions and recommendations:**

The prevalence of cerebral malaria was high among children with severe malaria. Factors associated with cerebral malaria included male gender, history of convulsions, abnormal bleeding, acute kidney injury, and hyponatremia. Targeted interventions and early management are essential to improve clinical outcomes.

**Supplementary Information:**

The online version contains supplementary material available at 10.1186/s12887-024-05178-z.

## Background

Malaria remains one of the most common and pernicious infectious diseases, despite being entirely preventable and treatable. In 2022, the global tally of malaria cases reached 249 million, with sub-Saharan Africa bearing the highest burden. Since 2015, malaria cases have increased globally, with a significant annual rise of 11 million cases between 2019 and 2020. Uganda remains a critical contributor to this increase, recording 597,000 additional cases between 2021 and 2022. The incidence in Uganda rose by 2%, reaching 267.8 cases per 1000 population at risk in 2022, underscoring its substantial role in the global malaria burden. Uganda bears the third-highest global burden of malaria, contributing 5.1% to the worldwide malaria cases, following Nigeria (26.8%), and D.R.Congo (12.3%) [1]. Children under the age of five are particularly vulnerable, contributing to more than 70% of all malaria-related mortalities in sub-Saharan Africa [2]. In Uganda, malaria accounts for approximately 13% of mortality in this age group [3]. Along with other common malaria syndromes, such as severe malarial anemia and respiratory distress (metabolic acidosis), cerebral malaria (CM) accounts for the majority of malaria-related deaths in African children [4]. Cerebral malaria (CM), primarily caused by *Plasmodium falciparum*, represents the most severe neurologic complication of malaria, particularly affecting children under five in sub-Saharan Africa [5, 6]. It is associated with high morbidity and mortality rates, particularly among young children [7]. A study conducted in Eastern Uganda by Olupot-Olupot et al*.* [8] reported an overall in-hospital mortality rate of 9.5% among children admitted with severe malaria, with cerebral malaria being the leading cause of death. This condition not only leads to immediate life-threatening complications but also long-term neurological and cognitive impairments in survivors. Besides the high mortality rate, cerebral malaria is responsible for serious neurological complications and other mental health issues, presumably as a result of ischemic neuronal damage [9, 10]. Furthermore, CM increases the risk of post-discharge hospitalizations related to malaria and other conditions [11].

Although the burden is acknowledged, there remains a substantial knowledge gap regarding the specific socioeconomic, environmental, and biological factors that predict CM in Ugandan children under five years [12]. Filling this gap is essential for creating targeted interventions and preventive measures, as current general malaria control strategies fail to effectively address CM's unique challenges [13]. While progress has been made in identifying risk factors for severe malaria syndromes like severe malarial anemia, the risk factors for CM, especially within Uganda's distinct epidemiological and socioeconomic context, are still poorly understood [14] [15]. This lack of focused research hinders the development of effective CM-specific interventions, leading to persistently high death rates and post-recovery complications among those who survive [16]. A literature survey indicates that while many studies have examined severe malaria broadly, research specifically analyzing CM's complexities in Uganda is limited, creating a significant gap in evidence-based policy and intervention strategies. Despite some notable advancements in understanding, more focused studies are needed to address this gap comprehensively [17]. This study aimed to determine the prevalence and factors associated with cerebral malaria among children aged 6 to 59 months who presented with severe malaria.

## Methods

### Study design, setting, and period

This was a descriptive and analytical hospital-based cross-sectional study. A quantitative approach was used to determine the prevalence and factors associated with cerebral malaria among hospitalized children aged 6 to 59 months with severe malaria in the pediatric ward and high dependency unit (HDU) of Fort Portal Regional Referral Hospital (FPRRH), located in the Tooro sub-region of western Uganda.

FPRRH serves as the regional referral hospital for nine districts, including Kabarole, Kamwenge, Kyegegwa, Kyenjojo, Bundibugyo, Ntoroko, Kasese, Bwamba, and Bunyagabu. It also receives patients from refugee camps in Kyegegwa, where 58.6% of children under the age of five are infected with malaria [18], as well as from the border area of the Democratic Republic of Congo (DRC), specifically from Ituri, a high transmission area for malaria. Tooro is among the top six regions contributing to over 60% of malaria deaths in Uganda. It is also one of the top ten regions contributing to over 50% of malaria cases in Uganda and covers moderate-to-high transmission areas of malaria. [19] [20]. FPRRH is a public regional referral facility under the Ministry of Health and serves as a Teaching Hospital affiliated with Kampala International University. The FRRH has a department of pediatrics and child health, offering a range of specialized services, including the pediatric ward and HDU, from which participants in this study were recruited. The pediatric ward has a capacity of seventy-five beds and is run by four pediatricians, three senior housing officers, and seven nurses. The pediatric ward at Fort Portal Regional Referral Hospital (FPRRH) has an average of 15 admissions per day, with nearly 9 of these admissions being children under the age of five. Among these, approximately 35% are admitted due to malaria. All cases of severe malaria were treated with intravenous (IV) artesunate, as recommended by the World Health Organization (WHO) guidelines for managing malaria cases. Data for this study were collected between January and March 2023.

### Study population

During the study period, all patients aged 6 to 59 months who were admitted to the pediatric ward and high-dependence unit of Fort Portal Regional Referral Hospital with severe malaria were considered eligible for inclusion.

### Selection criteria

This study included all children aged 6 to 59 months who were admitted with severe malaria as defined per the WHO criteria [4]. The same criteria were applied for identifying children with CM. Patients with conditions such as meningitis, encephalitis, epilepsy or cerebral vascular accidents in sickle cell disease were excluded from the study.

### Sample size determination and sampling technique

For the first objective (prevalence): The sample size was calculated using the Kish Leslie formula (1965): $n=\frac{{z}^{2}p\left(1-p\right)}{{e}^{2}}$; Where:

- n: the smallest number of participants needed
- p: assumed prevalence of the condition
- e: the 5% allowed error margin was set.
- z: (for the 95% confidence level) Z = 1.96).

Using the data from Olupot et al. (2020) [21], the calculation was: $\frac{{(1.96)}^{2}\times 0.074\times (1-0.074)}{{(0.05)}^{2}}=105$

Factors Associated with Cerebral Malaria: For the second objective, we used the modified Daniel’s formula to account for associated factors: $\text{n}=\frac{{\left({\text{Z}}_{1}+{\text{Z}}_{2}\right)}^{2}\times \frac{1}{\text{R}}\times \text{ P}(1-\text{P})}{{\text{e}}^{2}}$; Where:

- n = minimum sample size
- Z1 = 1.96 (95% confidence level)
- Z2 = 0.84 (80% power)
- R = odds ratio (3.0 based on Ibadin et al., 2012 [22]
- P = proportion of cerebral malaria among children using mosquito nets (31.2%)
- e = 5% margin of error

Using these values, the calculation was: $\text{n}=\frac{{\left(1.96+0.84\right)}^{2}\times \frac{1}{3}\times 0.312(1-0.312)}{{0.05}^{2}}=225$

To account for non-response and unfinished questionnaires, we added 10%:

$$\frac{n}{1-nr}=\frac{225}{1-0.1}= \frac{225}{0.9}=250$$

Therefore, the overall sample size was 250 children aged 6 to 59 months. Participants who met the eligibility criteria were consecutively enrolled until the required sample size was reached.

### Data collection procedures

All patients admitted to the Pediatrics ward and High Dependency Unit (HDU) of the Fort Portal RRH were screened for severe malaria according to the World Health Organization (WHO) criteria. Severe malaria was defined as the presence of asexual forms of Plasmodium falciparum in the blood, accompanied by one or more of the following: profound coma, severe anemia, respiratory distress, acidosis, acute kidney injury, circulatory collapse, spontaneous bleeding, multiple convulsions, hypoglycemia, or hemoglobinuria. Patients were assessed for the following clinical findings: severe pallor; convulsions; prostration, which was defined as the inability of children older than nine months to sit alone or to drink or breastfeed; and impaired consciousness, defined by a Blantyre coma score ≤ 2 in children. Clinically, shock was defined as cold extremities with a capillary refill > 3 s and a weak, fast pulse. Respiratory distress (acidotic breathing) was defined as deep, noisy, frequently fast breathing with increased inspiratory and expiratory chest exertion [4]. Jaundice, the yellowish discoloration of the skin and mucosal surfaces, was assessed clinically by checking the mucosal surfaces of the mouth and/or the sclera. Hemoglobinuria was diagnosed clinically in any child with a history of passage of dark-brown, tea-colored or Coca-Cola urine (according to a score of 5 or more on the Hillman urine color scale) during the present episode of illness observed by the caregiver and where possible by the study clinician. Abnormal bleeding was defined as spontaneous bleeding (defined as physically un-induced and irrepressible bleeding from at least two no traumatized sites in a patient with severe malaria without a previous history of abnormal bleeding). Cerebral malaria was defined as clinical syndrome characterized by coma ≥ 1 h after termination of a seizure or correction of hypoglycemia in the presence of asexual *Plasmodium falciparum* parasites on peripheral blood smears and no other cause to explain the coma. After identifying the study participants, clear explanations of the study procedures were given to them using Rutooro (a language well understood by the study participants). Subsequently, we obtained informed consent from those willing to participate. After completing the physical examination, 4 ml of blood was collected from each study participant, and the complete blood count (CBC), renal function test and serum electrolytes were studied. A peripheral thick blood smear was performed from a finger prick capillary blood sample. Laboratory factors, including malaria Hyperparasitemia, anemia, thrombocytopenia, acute kidney injury, hemoglobinuria, electrolyte imbalances (sodium and potassium) and hypoglycemia, were evaluated. In patients with a clinical suspicion of meningitis, a lumber puncture was performed to exclude meningitis or other central nervous system infections. All our study participants were treated according to the World Health Organization (WHO) and Uganda National Malaria Control Program guidelines. Upon admission, intravenous (IV) artesunate was administered as the first-line treatment for severe malaria. [23].

### Data collection instruments

A questionnaire was used to collect sociodemographic information such as the age of the child, education level of the caretaker, occupation of the caretaker, the residence, estimated monthly income of the parent or legal guardian and number of living children. The questionnaire also included information regarding health-seeking behaviors, including herbal medicine use**,** self-medication**,** and the time needed to seek health care facilities (delay in seeking health was considered when the time to arrive at the health facility exceeded 24 h after the onset of the illness). Malaria prevention practices, such as using insecticide-treated mosquito nets and controlling stagnant water around houses, were also considered in the questionnaire. The survey also included data related to medical factors such as duration of illness and immunization status. The nutritional status was also assessed using a UNICEF-designed, no stretchable MUAC, infantometer, stadiometer and weight scale [24]. A 5 ml syringe was used to draw blood. The blood was subsequently transported through a purple top vacutainer containing Ethylenediaminetetraacetic acid (EDTA) and a red top vacutainer without any additives. The complete blood count was analyzed using a Sysmex automated hematology analyzer (Sysmex X-N 550); renal function tests and serum electrolytes were analyzed using COBAS C311 S/N 1206/13 equipment. Blood sugar was measured using an “on call plus” glucometer.

### Malaria testing

The diagnosis of malaria was established through microscopic analysis. Initially, thick blood smears were prepared from capillary blood obtained via finger prick. Microscopic examination was conducted at magnifications of × 400 and × 1000 to determine the presence of *Plasmodium* parasites. For the quantification of parasitemia, the parasites observed were counted against a backdrop of 500 white blood cells (WBCs) under the × 100 objective. Malaria hyperpasitemia was defined as > 100,000 parasites/microliter.

### Quality assurance

Two pediatricians at Kampala International University Teaching Hospital (KIU-TH) reviewed the questionnaire and content validation form. They evaluated both the domains and items, assigning scores accordingly. The content validity index was calculated, with a score of at least 0.80 considered acceptable as recommended by Saiful & Yusoff (2019) [25]. A pilot study was subsequently conducted to pre-test the questionnaire and confirm its effectiveness. This pre-testing was carried out at Fort Portal Regional Referral Hospital, where ten questionnaires were administered. The feedback from this pilot study was used to refine the final questionnaire. Interviews were conducted in Rutooro, a language well understood by the study participants. All the instruments used in the study were calibrated and validated daily. The principal investigator continuously crosschecked the questionnaires, correcting errors during the data collection. Research assistants were adequately trained and routinely supervised by the principal investigator to ensure the correct use of the data tools and adherence to ethical principles. Blood smears were read by two laboratory technicians who were trained and validated by the Ministry of Health for reading blood smear slides.

### Data analysis

The data were entered into Epi-Data version 3.1 and exported to Windows’s Statistical Package for Social Sciences (SPSS) version 27 (Armonk. NY: IBM Corp) for all analyses. The prevalence of cerebral malaria was computed as the percentage of participants who had cerebral malaria relative to all participants with severe malaria. Logistic multivariate regression analysis was used to evaluate the associations between independent factors and cerebral malaria Multivariate analysis was performed if a variable had a *p* value ≤ 0.2 in the bivariate model. Both the crude and adjusted odds ratios with accompanying confidence intervals were reported. At the multivariate level, a *p* value ≤ 0.05 was considered to indicate statistical significance.

### Ethical considerations and consent

All procedures were implemented in strict adherence to the applicable national and international guidelines and regulations. The institutional Research and Ethics Committee of Kampala International University granted ethical approval (Reference No: KIU- 2022–218). The FPRRH management authorized the study to take place in the facility. Informed written consent was obtained from all parents or legal guardians.

## Results (Fig 1)

**Fig. 1:**
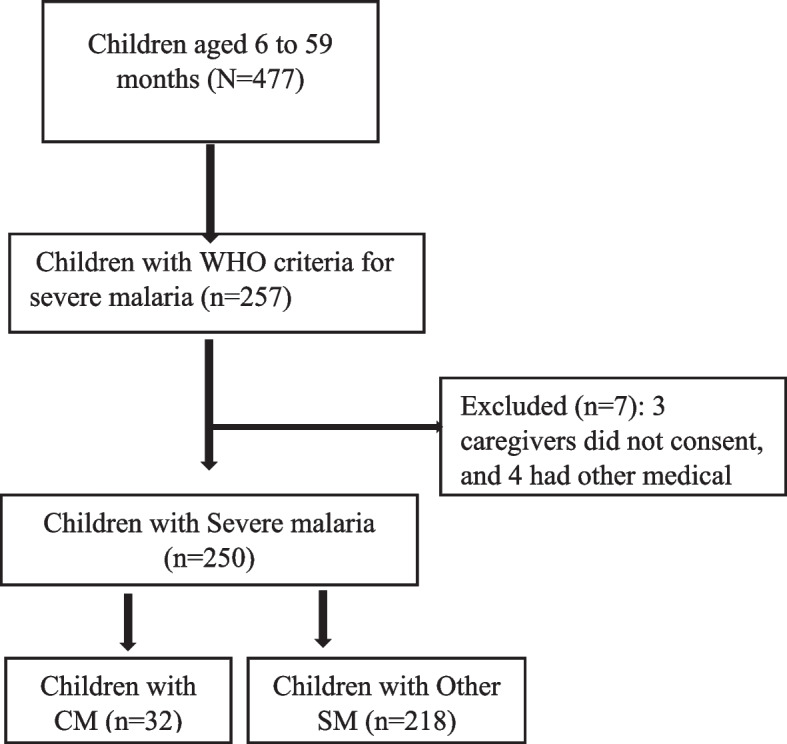
Study procedure

### Prevalence of cerebral malaria

The prevalence of cerebral malaria among children aged 6 to 59 months with severe malaria was 12.8%.

### Baseline demographic and socioeconomic characteristics of the study participants

The mean age of the study participants was 33.1 ± 17.3 months. The highest proportion of children (31.2%) were aged 48 to 59 months, and male and female participants were equally enrolled (50.8% vs 49.2%). Most parents/caretakers had attained a primary education (101/250, 40.4%) and earned a monthly income of less than 200,000 UGX equivalent to 57 US dollars (162/250, 64.8%). The majority of caregivers were married (192/250, 76.8%), and nearly two-thirds (66%) of the households had less than five children. Other demographic and socioeconomic characteristics are presented in Table 1.

**Table 1:** Demographic and socioeconomic characteristic of study participants

Variables	Frequency	Percent
Sex		
Male	127	50.8
Female	123	49.2
Age of children (months)		
< 12	41	16.4
12–23	38	15.2
24–35	43	17.2
36–47	50	20.0
48–59	78	31.2
Age of caretakers (years)		
16–25	78	31.2
26–35	105	42.0
36–45	57	22.8
46–55	10	4.0
Number of children per household		
< 5	165	66
≥ 5	85	34
Marital status		
Single	58	23.2
Married	192	76.8
Education		
Non formal	73	29.2
Primary	101	40.4
Secondary	71	28.4
Tertiary	5	2
Occupation		
Non formal	224	89.6
Formal	26	10.4
Religion		
Christians	226	90.4
Muslim	16	6.4
Others	8	3.2
Monthly income (UGX)		
< 200 K	162	64.8
≥ 200 K	88	35.2

*UGX* Ugandan shilling, Formal occupation = caretakers who earned salaries while working (private or public sectors). Non-formal occupation = self-employed caretakers with no salaries (peasants, small business holders, street vendors, small-scale artisans, and informal traders)

### Health-related behavior and malaria prevention practices of the study participants

Most caretakers reported self-medication (174/250, 69.6%), and the majority of patients delayed arriving at the hospital after the onset of illness (239/250, 95.6%). The use of insecticide-treated bed nets was reported by 67.6% (169/250) of the study participants. The other health-related characteristics and malarial prevention practices of the study participants are presented in Table 2.

**Table 2:** Health-related behavior and malarial prevention practices of study participants

Variables	Frequency	Percent
Use of herbal medicine		
Yes	97	38.8
No	153	61.2
Self-medication		
Yes	174	69.6
No	76	30.4
Delay in seeking health facility		
Yes	239	95.6
No	11	4.4
Use of mosquito net (LLINs)		
Yes	169	67.6
No	81	32.4
Presence of stagnant water		
Yes	140	56
No	110	44

*LLINs* Long Lasting Insecticidal Nets

### Clinical and laboratory characteristics of severe malaria among study participants

Approximately one-third of participants had a history of convulsions (82/250, 32.8%) and presented with hemoglobinuria (83/250, 33.2%). Most of the study participants had prostration (189/250, 75.6%). Hyperparasitemia (204/250, 81.6%), thrombocytopenia (110/250, 44%), and severe anemia (80/250, 32%) were the most common laboratory findings (Table 3*)*.

**Table 3:** Clinical and laboratory characteristics of severe malaria among study participants

Variables	frequency	Percentage
History of convulsion		
Yes	82	32.8
No	168	67.2
Hemoglobinuria		
Yes	83	33.2
No	167	66.8
Shock		
Yes	6	2.4
No	244	97.6
Jaundice		
Yes	44	17.6
No	206	82.4
Abnormal bleeding		
Yes	7	2.8
No	243	97.2
Hyperparasitemia		
Yes (> 100,000 parasites/μL)	204	81.6
No (< 100,000 parasites/μL)	46	18.4
Hypoglycemia		
Yes	57	22.8
No	193	77.2
Hemoglobin level (g/dl)		
Severe anemia **(**< 7)	74	29.6
Moderate anemia (7 to < 9)	78	31.2
Mild anemia (9–11)	82	32.8
No anemia (> 11)	16	6.4
Thrombocytopenia		
Yes	110	44
No	140	56
Acute kidney injury		
Yes	48	19.2
No	202	80.8
Hyponatremia		
Yes	68	27.2
No	182	78.8
Hypernatremia		
Yes	8	3.2
No	242	96.8
Hypokalemia		
Yes	44	17.6
No	206	82.4
Hyperkaliemia		
Yes	25	10
No	225	90

*Parasites/μL* parasites per microliter

### Factors associated with cerebral malaria

Based on the results of bivariate and multivariate analyses (Table 4), the following factors were significantly and independently associated with cerebral malaria. The likelihood of cerebral malaria was 3.05 times greater in males than in females (95% C. I: 1.20–7.77, *p* = 0.020). The odds of cerebral malaria were 12.20 times greater among children with a history of convulsion than among those without convulsions (95% C. I: 10.7–21.69, *p* = 0.030). There were 13.22 abnormal bleeding events (95% C. I: 11.54–15.16) more likely to have cerebral malaria than did those without abnormal bleeding. There were 4.50-fold greater incidences of acute kidney injury and hyponatremia (95% C. I: 1.30–15.53, *p* = 0.020) and 3.47 times (95% C. I: 1.34–8.96, *p* = 0.010) more likely to develop cerebral malaria than other children.

**Table 4:** Factors associated with cerebral malaria among children aged 6 to 59 months with severe malaria

Variables	Cerebral malaria		cOR (95% C.I)	aOR (95% C.I)
	**Yes (%)**	**No (%)**		
Sex				
Male	23(71.9)	104(47.7)	2.80(1.40–6.33)	3.05(1.20- 7.77) *
Female	9(28.1)	114(52.3)	1	1
History of convulsion				
Yes	30(93.8)	52(23.9)	17.88(11.07–20.71)	12.20(10.7- 21. 69) *
No	2(6.2)	166(76.1)	1	1
Hemoglobinuria				
Yes	7(21.8)	76(34.9)	1.91(0.79–4.62)	0.20(0.05- 1.81)
No	25(78.2)	142(65.1)	1	1
Abnormal bleeding				
Yes	4(12.5)	3(1.4)	10.24(2.18–28.13)	13.22(11.54- 15.16) *
No	28(87.5)	215(98.6)	1	1
Hyperparasitemia				
Yes	29(90.7)	175(80.2)	2.38(0.69–8.16)	2.56(0.60- 11.01)
No	3(9.3)	43(19.8)	1	1
Acute kidney injury				
Yes	10(31.2)	38(17.4)	2.15(0.94–4.91)	4.50(1.30- 15.53) *
No	22(68.8)	180(82.6)	1	1
Hyponatremia				
Yes	19(59.3)	49(22.4)	3.71(1.73–7.95)	3.47(1.34- 8.96) *
No	13(40.7)	167(76.6)	1	1

*AOR* adjusted odds ratio, *cOR* crude odds ratio, *CI* confidence interval

^*^statistically significant (*p* < 0.05)

## Discussion

The prevalence of cerebral malaria was 12.8% (32/250, 95% CI = 8.9%-17.6%). This prevalence is higher than that reported in Eastern Uganda (1.4%) by Olupot-Olupot et al. in 2020 [8]. This difference was due to the different study populations. Olupot-Olupot et al. included children aged between 2 months and 12 years. Studies have demonstrated that malaria is less common among infants younger than 6 months old [26, 27], while those older than five years have a low risk of severe disease [8]. On the other hand, our findings are comparable to those of other studies conducted in East Africa and other sub-Saharan countries, where the prevalence of cerebral malaria varies between 9.2% and 16.6% [28–30]. In contrast, the prevalence reported in this study was lower than that reported in studies conducted in Ghana [31], the Democratic Republic of Congo [32] [33], and Gabon [34], where the prevalence was 23%, 30.1% to 48.4%, and 75.4%, respectively. Differences in prevalence may be related to variations in the sociodemographic characteristics of the study participants, such as age [8, 32], and variations in the criteria for the diagnosis of cerebral malaria [34]. The current study used the WHO definition of cerebral malaria [4] and was less likely to include patients in a postictal state or with other metabolic complications; however, all the above studies defined CM as children whose comatose state progressed in less than 30 min and/or had at least two episodes of generalized seizure within 24 h and/or who were in a convulsive status. This last definition may include more patients than the one we used.

Factors that were independently associated with cerebral malaria among children with severe malaria were male sex, abnormal bleeding, history of convulsions, acute kidney injury, and hyponatremia.

In this study, male and female participants were almost equally represented (50.8% male vs. 49.2% female). However, males had a 3.05-fold increased likelihood of developing cerebral malaria (CM) compared to females. This observed gender disparity in CM incidence raises important questions about the underlying factors contributing to this difference. The relationship between CM and male sex in children has yielded mixed results in various studies. Some research suggests that boys may be at a slightly greater risk of severe malaria, including CM, potentially due to differences in immune responses, genetic factors, or exposure risks [27]. Additionally, females typically develop more robust immune responses against parasites than males and can clear parasites faster in cases of asymptomatic malaria [35, 36]. Hormonal differences, such as testosterone's immunosuppressive effects and estrogen's immune-enhancing properties, might also play a role. Behavioral factors, including differing exposure risks due to outdoor activities, and genetic variations in immune response genes, could contribute as well [35, 37]. Conversely, other studies have found no significant association between sex and the incidence of cerebral malaria [38, 39]. The current evidence remains inconclusive, highlighting the need for further research to comprehensively understand the sex-specific risks and underlying mechanisms associated with cerebral malaria.

Children presenting with abnormal bleeding exhibited a 13.22-fold increased risk of developing cerebral malaria. The reported incidence of bleeding in severe malaria patients varies considerably from less than 10% to 25% [40]. Although overt bleeding is rarely reported in the pediatric population, it occurs in approximately 5% of children with cerebral malaria and most commonly from the gastrointestinal tract [40, 41]. Cerebral malaria is a multisystemic pathology, and organ damage could explain bleeding disorders associated with cerebral malaria [42]. Blood coagulation activation frequently occurs in patients with severe malaria, and clinically apparent bleeding or disseminated intravascular coagulation is associated with very severe diseases such as cerebral malaria [41]. Bleeding abnormalities and coagulopathy have been reported among patients with non-neurologic complications and concurrent manifestations of cerebral malaria [42]. While gross hemorrhagic or thrombotic events are uncommon in cerebral malaria, their occurrence highlights a clear interaction between P. falciparum infection and coagulation [43, 44]. This finding highlights the importance of recognizing abnormal bleeding as a potential indicator of cerebral malaria in children under the age of five.

Children with convulsions were 12.20 times more likely to have cerebral malaria. These findings were in agreement with previous studies reporting a correlation between multiple convulsions at admission and the occurrence of cerebral malaria [9]. In Ugandan children, a history of convulsions was an independent risk factor associated with impairment of consciousness in children with severe malaria [34, 45–47]. Convulsions in the presence of malaria are more frequently a sign of cerebral dysfunction than just being febrile fits [48]. The causes of seizures are unclear; most are not associated with fever [49] or electrolyte disorders [50]. Several studies have suggested that ischemia and hypoxia may play a part in this process and that seizures might be caused by sequestration of infected erythrocytes or parasite-derived toxins [51]. Furthermore, immune mechanisms may be important because children with severe malaria and seizures have high titers of antibodies to voltage-gated calcium channels [52].

In the present study, children with acute kidney injury (AKI) exhibited a 4.5-fold increased likelihood of developing cerebral malaria compared to those with normal renal function. This result is in agreement with the findings of a cohort study conducted in Ugandan children aged between 18 months and 12 years evaluating cerebrospinal fluid biomarkers for kidney-brain axis involvement in cerebral malaria pathogenesis, where 46.3% of those with cerebral malaria also had acute kidney injury [46]. In a retrospective case‒control observational study conducted in Zambia involving children aged between 2 and 11 years with central nervous system malaria, 134 out of 209 (64.1%) study participants had AKI according to the KDIGO criteria [53]. In a prospective cohort study in India, cerebral malaria was found to be an important risk factor for mortality in malarial AKI patients [54]. Cerebral malaria occurs as a multisystemic pathology [42]. In Ugandan children, multi-organ dysfunction was common, with 76.2% of children having at least one organ system affected in addition to coma [46]. Cerebral malaria and AKI arise due to vesicular damage resulting from immunological reactions [55]. In patients with cerebral malaria, kidney dysfunction and acidosis are independent predictors of mortality, both in children and adults [56]. The pathogenic mechanisms of AKI in cerebral malaria have not yet been fully defined but include parasite sequestration, endothelial dysfunction, oxidative stress, and immune-mediated damage [53]. In children with cerebral malaria, there is evidence of kidney-brain injury, and multiple potential pathways have been identified [46].

Children with hyponatremia were 3.47 times more likely to have cerebral malaria. Other studies have reported that more than half of patients with CM also experience hyponatremia [47, 57]. The pathophysiology of hyponatremia in cerebral malaria remains unclear, but several studies have suggested that increased secretion of vasopressin plays an important role [58]. Hyponatremia in cerebral malaria may also result from mechanisms such as syndrome caused by inappropriate antidiuretic hormone secretion, cerebral salt wasting, or gastrointestinal and renal losses [59]. A high ADH concentration might be a marker of a decrease in the effective plasma volume, suggesting TNF-alpha-induced endothelial damage and leakage [60]. However, dehydration, more than SIADH, was suggested to be the cause of hyponatremia in severe childhood malaria [50].

To the best of our knowledge, this is the first study to assess the factors associated with cerebral malaria among children in Uganda. This study has several limitations; the study was conducted at a single regional referral hospital, which may limit the generalizability of the findings to other regions with different demographic and epidemiological profiles. As a cross-sectional study, it captures data at a single point in time, which restricts the ability to infer causation or observe temporal trends. The reliance on hospital records and self-reported data by parents or guardians may introduce recall bias or inaccuracies in reporting.

The present study reported a high prevalence of cerebral malaria among children with severe malaria. Factors associated with cerebral malaria were male sex, a history of convulsions, and other features of severe malaria, such as abnormal bleeding, acute kidney injury, and hyponatremia. Clinical and laboratory manifestations of severe malaria should be promptly investigated and adequately treated to reduce the risk of cerebral malaria in young children.

## Supplementary Information

Supplementary Material 1.

## Data Availability

Data is available upon request. Requests should be sent to bangamseza@gmail.com (BM).

## Abbreviations

aOR: Adjusted odd ratio
ADH: Antidiuretic hormone
AKI: Acute kidney injury
CI: Confidence interval
cOR: Crude odd ratio
CBC: Complete blood count
CM: Cerebral malaria
DRC: Democratic Republic of Congo
FPRRH: Fort Portal Regional Referral Hospital
HDU: High-Dependent Unit
KDIGO: Kidney Disease Improving Global Outcomes
KIU: Kampala International University
IBM SPSS: Statistical Package for the Social Sciences
MUAC: Mid-upper arm circumference
TNF: Tumor necrosis factor
SIADH: Syndrome of Inappropriate Antidiuretic Hormone Secretion
UGX: Uganda shillings
UNICEF: United Nations International Children’s Emergency Fund
WHO: World Health Organization
