# Supplementary material for: Prevalence and factors associated with cerebral malaria among children aged 6 to 59 months with severe malaria in Western Uganda: a hospital-based cross-sectional study
Source: BMC Pediatr. 2024 Nov 6;24:704. doi: 10.1186/s12887-024-05178-z (PMC11539429; doi:10.1186/s12887-024-05178-z)
Supplement: Supplementary file 1 — Supplementary Material 1. [file 12887_2024_5178_MOESM1_ESM.pdf]

Appendix VI (A): Questionnaire (English version)

Title: "Prevalence and factors associated with cerebral malaria amongst hospitalized children 6 to 59 months with severe malaria at Fort Portal Regional Referral Hospital, Western Uganda"

Respondent's ID number. ……………….. Date…………….……

SECTION A: SOCIODEMOGRAPHIC DATA

1. Address ……………………………………………………………………………………………
2. Age of the child (in months) ……………………Date of birth …………………………..
3. What is the sex of your child?

   Male ☐ Female ☐
4. What is the tribe of your child?

Kabarole☐ Kyenjojo ☐ Bunyangabo ☐ Munyankole ☐ Other (specify)

SECTION B: DATA OF THE MOTHER

5. Date of birth …………………………Age (years) ………………………………
6. Level of education: No formal education ☐ Primary ☐ Secondary ☐ Tertiary ☐
7. Occupation: ……………………… Tribe……………………. Religion………………………
8. Number of living children…
9. Marital status: Single ☐ Married ☐ Widow ☐ Divorced ☐
10. Monthly income: Less than 200,000 ☐ 200,000 to 500, 000 ☐ More than 500,000 ☐

SECTION C: MEDICAL DATA

5. For how long your child has been unwell?

   <24 Hour ☐ 24- 48 Hour ☐ 48- 72 Hours ☐ > 72 hours ☐

6. When did you reach the hospital?…………………………………………………………………

   After 1 days ☐ between 2-3 days ☐ between 3-4 days ☐ ≥ 5 days
7. What is the immunization status of your child?

   Up-to-date ☐ Not up-to-date ☐ Never immunized ☐

8. Did you visit any other health facility (Clinic or health center) before coming in FPRRH?

   Yes ☐ No ☐

KIU RESEARCH ETHICS COMMITTEE(REC)
APPROVED
VALID UNTIL
10 JAN 2024
P.O. Box 71, Bushenyi - Ishaka

9. Did you use any herbal medicine before reaching health facility?

Yes ☐ No ☐

10. Did you visit any patent medicine vendors before seeking the health facility?

Yes ☐ No ☐

11. Did you use any anti- malaria drug before reaching the hospital

Yes ☐ No ☐

12. Do you use insecticides treated mosquito- net at home?

Yes ☐ No ☐

13. There is any presence of stagnant water around your house (or creek)?

Yes ☐ No ☐

14. Did your child convulsed before reaching hospital?

Yes ☐ No ☐

15. What is the colour of the urine of your child?

SECTION C: PHYSICAL EXAMINATION

16. Vital signs: RR:............... PR: .... T°..................CRT:..........

17. Patient in Shock?

Yes ☐ No ☐

18. Level of consciousness (Blantyre Paediatric Score):

....

19. Prostration?

Yes ☐ No ☐

20. Presence of jaundice?

Yes ☐ No ☐

21. Palpable liver?

Yes ☐ No ☐

22. Palpable spleen?

Yes ☐ No ☐

23. Abnormal bleeding

Yes ☐ No ☐

24. Urine output in 24 hours period?

....

KIU RESEARCH ETHICS COMMITTEE(REC)
APPROVED
VALID UNTIL
18 JAN 2024
KIU
P.O. Box 71, Bushenyi - Ishaka

Scanné avec CamScanner

25. Color of the urine?

……………………………………..

26. Acute kidney injury?

Yes ☐ No ☐

SECTION D: LABORATORY DATA

Hyperparasitemia	RBS	Hemoglobin	WBC	PLT	ELECTROLYTE
Yes ☐<br>No ☐	Normal ☐<br>Hypoglycemia ☐<br>hyperglycemia ☐	Normal ☐<br>Anemia ☐<br>Severe anemia ☐	Normal ☐<br>Leucopenia ☐<br>Leukocytosis ☐	Normal ☐<br>Thrombocytopenia ☐<br>Thrombocytosis ☐	Sodium<br>Normal ☐ hyper ☐ hypo ☐<br>Potassium:<br>Normal ☐ hyper ☐ hypo ☐

KIU RESEARCH ETHICS COMMITTEE(REC)
APPROVED
VALID UNTIL
18 JAN 2024
KIU
P.O. Box 71, Bushenyi - Ishaka

Scanné avec CamScanner
